# Utilizing extracorporeal membrane oxygenation and surfactant in the management of severe acute respiratory distress syndrome due to hydrocarbon pneumonitis

**DOI:** 10.1177/02676591221148605

**Published:** 2022-12-22

**Authors:** Christina R Rufener, Nathan A Friedman, Jordan E Vaught, Helen A Harvey, Nicole G Coufal

**Affiliations:** 1 Department of Pediatrics, 8784 University of California at San Diego, La Jolla, CA, USA; 2 Rady Children’s Hospital San Diego, San Diego, CA, USA; 3 Department of Emergency Medicine, Division of Medical Toxicology, 8784 University of California, San Diego, CA, USA

**Keywords:** Acute respiratory distress syndrome, drug overdose, mechanical ventilation, surfactant, extracorporeal membrane oxygenation

## Abstract

Severe cases of hydrocarbon aspiration requiring Extracorporeal Membrane Oxygenation (ECMO) are rarely reported in pediatrics, and 90% of hospitalized patients have a relatively benign clinical course. We describe a 14 month-old female with accidental hydrocarbon ingestion and aspiration due to organic makeup brush cleaner that suffered severe ARDS and multiorgan failure, successfully managed with ECMO and surfactant. She was decannulated after a total of 72 hours on ECMO, extubated on hospital day 15 (HD 15), and discharged home in her normal state of health after one month in the hospital. ECMO and adjunctive therapies such as surfactant may be helpful in the management of severe hydrocarbon pneumonitis and there are limited reports of ECMO as a supportive method for these pediatric patients.

## Case report

**Figure 1.**
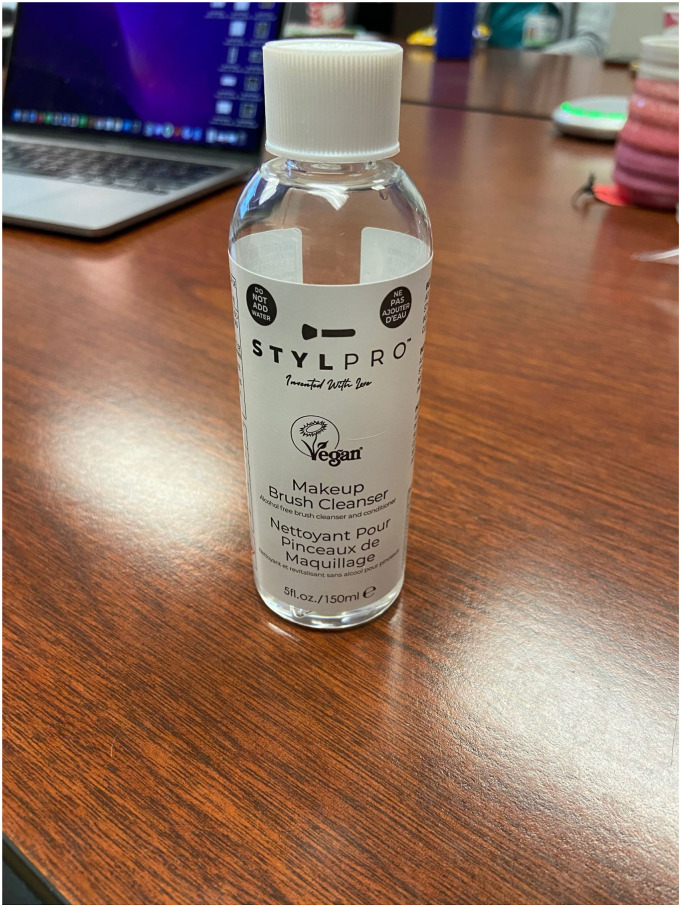
Bottle of the makeup brush cleaner.

**Figure 2.**
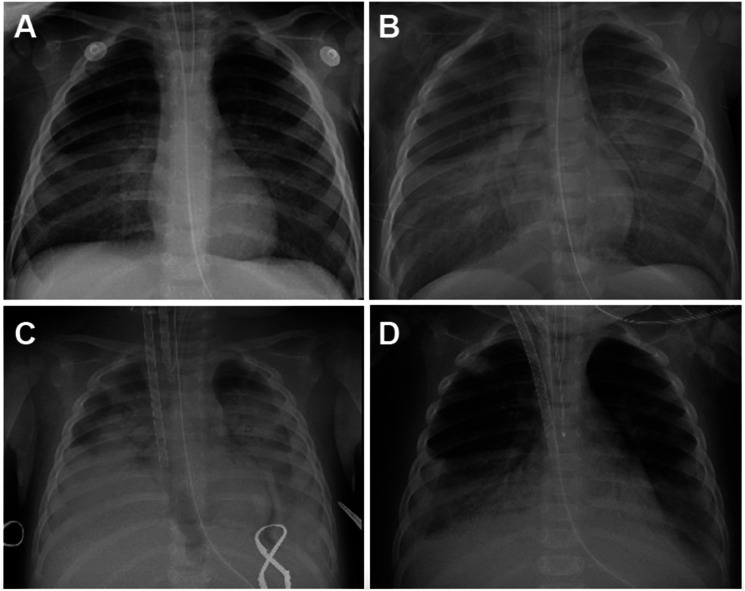
Progression of chest radiographs through intubation and ECMO cannulation.

A previously healthy 14 month-old female presented with altered mental status, cough, and cyanosis. She was found with an open bottle of “organic makeup brush cleaner” (Figure 1) with cleaner covering her lips, face, and hands. She immediately started coughing and became somnolent with peri-oral cyanosis. Her father then induced vomiting by digital stimulation and her mental status worsened with intermittent apnea. This prompted transport to the ED where she was intubated for lethargy and acute respiratory failure (Figure 2(a)). The makeup brush cleaner contained “>30 aliphatic hydrocarbons” with only 1 ingredient specifically detailed - 2,2,4,4,6,8,8-heptamethylnonane, confirmed later by GC/MS as the sole ingredient.

In the pediatric intensive care unit she rapidly developed severe ARDS with an oxygenation index (OI) of 27. Despite inhaled nitric oxide (40 ppm), proning, and paralysis, her OI increased to 56 with worsening bilateral opacities (Figure 2(b)). She developed shock refractory to vasopressors and high-dose steroids.

Her hospital course was further complicated by a fulminant systemic inflammatory response, with large bilateral pleural effusions, ascites, and abdominal compartment syndrome. Her fluid overload required hemofiltration, diuretics, and peritoneal drain placement. An echocardiogram demonstrated reduced biventricular systolic function and left ventricular ejection fraction of 18%. Due to refractory shock and hypoxia, she was cannulated onto veno-arterial (VA) ECMO on HD 1 (Figure 2(c)) requiring maximum support with a cardiac index (CI) of 2.5 L/min/m^2^. On HD 2, she received a single dose of surfactant (Curosurf) 2.5 mL/kg by endotracheal lavage with an immediate decrease in ECMO support need from a CI of 2.5 to 2.0 L/min/m^2^ and a 10% reduction in ECMO FiO2 need within 2 hours. The peak inspiratory pressure (PIP) was also able to be weaned within an hour of surfactant administration to maintain the same tidal volumes on the ventilator. She was successfully decannulated after 72 hours of ECMO (Figure 2(d)).

**Figure 3.**
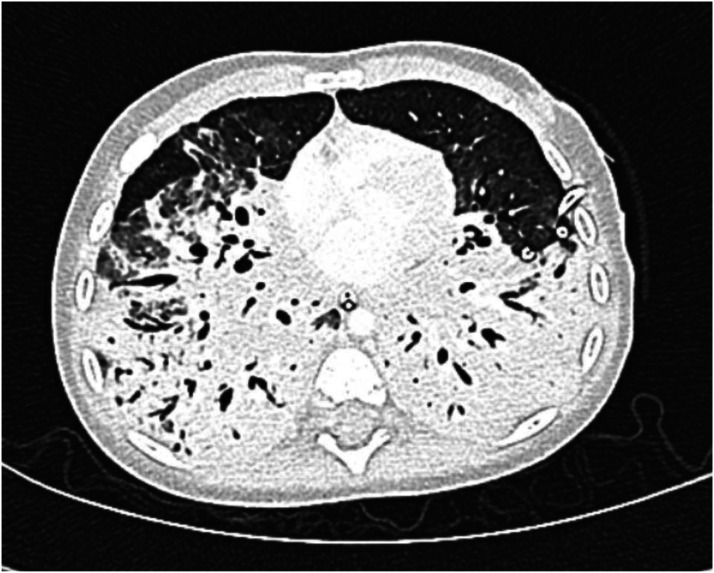
Chest CT on hospital day 11 demonstrating bilateral lower lobe lung necrosis.

Her hospital course was subsequently complicated by spontaneous pneumothorax requiring tube thoracostomy. A chest CT demonstrated extensive areas of lung necrosis (Figure 3). She was successfully extubated on HD 15, weaned to room air on HD 25, and discharged home on HD 35 without respiratory support and in her usual state of health.

## Discussion

Hydrocarbons are a common and potentially lethal component in numerous household items.^1^ They are primarily manufactured for use as a fuel source, however also have widespread use in paint thinners and other petroleum distillates such as furniture polishes, solvents, and cleaners.^2^ The clinical course of accidental hydrocarbon aspiration or inhalation is usually mild with coughing, vomiting, and central nervous system depression as common presenting symptoms.^2^ Severe cases of a rapid chemical pneumonitis, ARDS, and multiorgan failure are more classically described with exposure to kerosene and paint thinner exposures. There are no known demographics or clinical factors that are significant prognostic indicators of disease severity.^2^ The severity of the clinical course in this case is unique due to what is generally thought of as a more innocuous household source - an organic makeup brush cleaner.

After entering the lung, hydrocarbons destroy surfactant and directly damage lung parenchyma, leading to a progressive chemical pneumonitis, necrotizing bronchopneumonia, and refractory hypoxia.^3^ Studies have documented detrimental long term pulmonary effects of hydrocarbon pneumonitis which lead to chronic lung disease in adulthood.^4^ Cardiac dysrhythmias are also reported,^2^ particularly with aromatic or halogenated hydrocarbons causing sensitization of the myocardium to catecholamines.

There is a large variation in the duration of ECMO therapy for severe hydrocarbon pneumonitis with most cases requiring between 1 to 4 weeks.^3–5^ The duration of ECMO required in this patient was brief in comparison, potentially due to the administration of surfactant. Although there is limited data on the role of exogenous surfactant in hydrocarbon pneumonitis, it is reasonable to postulate that it may be beneficial in patients with severe pneumonitis due to the direct destructive effects of hydrocarbons on surfactant. There are six previously reported cases in which surfactant was used as an adjunct in hydrocarbon aspiration (Table 1), only one of which was also in the setting of ECMO cannulation.^9,10^ The preferred dose and preparation of surfactant for acute lung injury has largely been based on a multi-center randomized control trial by Willson et al.^5^ which specifically utilized Calfactant. Calfactant has higher levels of hydrophobic surfactant-specific-protein B conferring greater resistance to proteins associated with lung injury and thus equal activity to natural surfactant. In their study, a significant decrease in mortality and improvement in OI was seen in children receiving Calfactant compared to the placebo group. Early administration of surfactant in hydrocarbon pneumonitis is also preferred due to emerging evidence in literature demonstrating rapid and direct improvement of patient gas exchange values.^8^ After administration of surfactant, this patient similarly exhibited significant improvement within 24 hours. There are no reported negative effects following surfactant administration in hydrocarbon aspiration. In this case, surfactant appeared to act as a beneficial adjunct to ECMO.

**Table 1.** Reported cases of surfactant use in hydrocarbon pneumonitis.

Source	Age	Agent	Dose of surfactant	Timing of surfactant after ingestion	Use of ECMO	Duration of mechanical ventilation	Hospital LOS
Horoz et al ^6^	17 months	Cleaning naphta	8 mL/kg Survanta, two doses separated by 12 hours	3 days	No	Unknown	Unknown. PICU LOS 18 days
Sommer et al ^7^	17 months	Citronella candle fluid	3.5 mL/kg calfactant	18 hours	No	6 days	11 days
Sommer et al ^7^	19 months	Tiki torch fluid	3 mL/kg calfactant	Unknown	No	60 hours	10 days
Mastropietro and Valentine^8^	19 months	Lamp oil	3 mL/kg calfactant, two doses separated by 9 hours	10 hours	No	64 hours	Unknown. PICU LOS <5 days
Richards et al ^9^	18 months	Tea tree oil	3 mL/kg unspecified “surfactant”	1 hour	No	2 days	3 days
Willaert et al ^5^	18 months	Oil-based cosmetic	2 mL/kg survanta	60 hours	Yes, VV-ECMO	14 days	Unknown
Current case	14 months	Makeup brush cleaner	2.5 mL/kg curosurf	40 hours	Yes, VA-ECMO	15 days	35 days

## Conclusion

The use of ECMO remains a rare, yet promising therapy in recovery from severe ARDS due to hydrocarbon aspiration. Surfactant as an adjunctive therapy should be considered early in severe intoxications and it may decrease the duration of ECMO, although further studies are needed. This case highlights the development of severe ARDS and multiorgan failure following an ingestion of an aliphatic hydrocarbon-based makeup brush cleaner, requiring the shortest reported ECMO course possibly facilitated by the addition of surfactant.

## References

[1] Makrygianni EA Palamidou F Kaditis AG . Respiratory complications following hydrocarbon aspiration in children. Pediatr Pulmonol 2016; 51(6): 560–569. DOI: 10.1002/ppul.23392. 26910771

[2] Tenenbaum A Rephaeli R Cohen-Cymberknoh M , et al. Hydrocarbon intoxication in children: clinical and sociodemographic characteristics. Pediatr Emerg Care 2021; 37(10): 502–506. DOI: 10.1097/PEC.0000000000002111. 32433458

[3] Chyka PA . Benefits of extracorporeal membrane oxygenation for hydrocarbon pneumonitis. J Clin Toxicol 1996; 34(4): 357–363. DOI: 10.3109/15563659609013804. 8699548

[4] Bille AB Pedersen KD Hertel S . Extracorporeal membrane oxygenation of a child with severe chemical pneumonia. Ugeskrift for Laeger 2011; 173(48): 3115–3116. 22118656

[5] Willaert A Orozco B Maslonka K , et al. Surfactant administration as treatment for respiratory failure following hydrocarbon aspiration. Clinical Toxicology 2013; 51: 590–591.

[6] Horoz OO Yildizdas D Yilmaz HL . Surfactant therapy in acute respiratory distress syndrome due to hydrocarbon aspiration. Singapore Med J 2009; 50(4): 130–132. 19421666

[7] Sommer C Johnson AB Sam Wang G , et al. Surfactant for the management of pediatric hydrocarbon ingestion. Am J Emerg Med 2018; 36(12): 2260–2262. DOI: 10.1016/j.ajem.2018.09.016. 30236893

[8] Mastropietro CW Valentine K . Early administration of intratracheal surfactant (calfactant) after hydrocarbon aspiration. Pediatrics 2011; 127(6): 1600–1604. DOI: 10.1542/peds.2010-3229. 21624880

[9] Richards DB Wang GS Buchanan JA . Pediatric tea tree oil aspiration treated with surfactant in the emergency department. Pediatr Emerg Care 2015; 31(4): 279–280. PMID: 25285387. DOI: 10.1097/PEC.0000000000000234. 25285387

[10] Willson DF Thomas NJ Markovitz BP , et al. Effect of exogenous surfactant (calfactant) in pediatric acute lung injury: a randomized controlled trial. JAMA 2005; 293(4): 470–476. DOI: 10.1001/jama.293.4.470. 15671432

